# The Physicians’ Visiting List for 1891

**Published:** 1891-01

**Authors:** 


THE PHYSICIAN'S VISITING LIST FOR 1891. 40th year of its publication. Philadelphia: P. Blakiston, Son & Co.

The Physician's Visiting List of P. Blakiston Son & Co. is one of the most convenient and valuable pocket books which the practitioner can have. Its tables of almanac, poison and antidotes, weights and measures, dose table, list of new remedies, page on disinfectants, examination of urine, methods for assisting respiration, etc., are as accurate and convenient as condensed space allows them to be. The list of patients for daily visits, memoranda, engagements and cash account are nicely divided to answer all ordinary purposes. While it is a physician's note book, it is one of the best adapted for veterinary practitioners.

